# Affinity Tag-Free Purification of SARS-CoV-2 N Protein and Its Crystal Structure in Complex with ssDNA

**DOI:** 10.3390/biom14121538

**Published:** 2024-11-30

**Authors:** Atanu Maiti, Hiroshi Matsuo

**Affiliations:** Cancer Innovation Laboratory, Frederick National Laboratory for Cancer Research, Frederick, MD 21702, USA

**Keywords:** COVID-19, SARS-CoV-2, nucleocapsid protein, protein expression, protein purification, mass photometry, N-terminal domain, X-ray crystallography, atomic-resolution structure, protein–DNA interactions

## Abstract

The nucleocapsid (N) protein is one of the four structural proteins in SARS-CoV-2, playing key roles in viral assembly, immune evasion, and stability. One of its primary functions is to protect viral RNA by forming the nucleocapsid. However, the precise mechanisms by which the N protein interacts with viral RNA and assembles into a nucleocapsid remain unclear. Compared to other SARS-CoV-2 components, targeting the N protein has several advantages: it exhibits higher sequence conservation, lower mutation rates, and stronger immunogenicity, making it an attractive target for antiviral drug development and diagnostics. Therefore, a detailed understanding of the N protein’s structure is essential for deciphering its role in viral assembly and developing effective therapeutics. In this study, we report the expression and purification of a soluble recombinant N protein, along with a 1.55 Å resolution crystal structure of its nucleic acid-binding domain (N-NTD) in complex with ssDNA. Our structure revealed new insights into the conformation and interaction of the flexible N-arm, which could aid in understanding nucleocapsid assembly. Additionally, we identified residues that are critical for ssDNA interaction.

## 1. Introduction

The COVID-19 pandemic, caused by Severe Acute Respiratory Syndrome Coronavirus-2 (SARS-CoV-2), is one of the most devastating health and socioeconomic crises in modern history [1,2,3]. According to the World Health Organization (WHO), approximately 777 million people have been infected, and 7.1 million people have died due to SARS-CoV-2 infection to date (https://data.who.int/dashboards/covid19/cases). Although the timely development and worldwide implementation of vaccines have helped contain the pandemic, newly emerging variants still pose threats to human health and present risks for future pandemics [4,5]. Therefore, efforts to better understand SARS-CoV-2 and to develop antiviral agents have been ongoing since the pandemic began [6,7,8].

**Figure 1:**
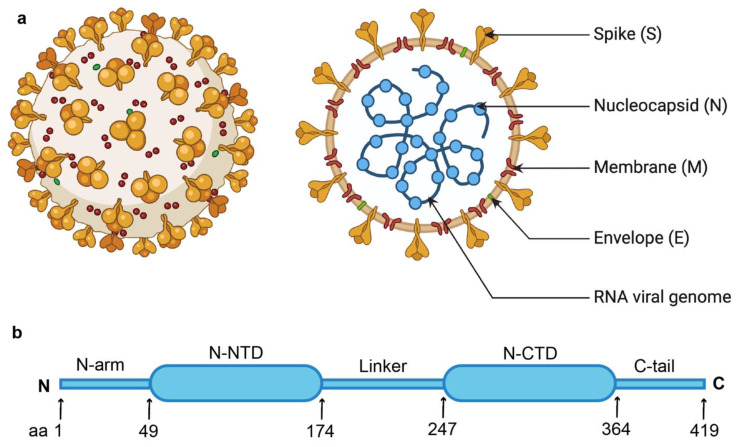
(**a**) Schematic representation of SARS-CoV-2 virus and its structural proteins (S, N, M, and E) with genomic RNA [created with BioRender.com]. (**b**) The N protein is composed of several distinct regions: the N-terminal domain (N-NTD) and the C-terminal domain (N-CTD) connected by a flexible linker, and intrinsically disordered regions like the N-arm and the C-tail.

SARS-CoV-2 is a positive-sense single-stranded RNA (ssRNA) virus that belongs to the betacoronavirus family and shares genetic and structural homology with SARS-CoV (which caused the 2002 SARS epidemic) and MERS-CoV (which emerged in 2012) [9,10]. The large RNA genome (approximately 30 kb) of SARS-CoV-2 encodes 16 non-structural proteins (NSPs) and at least six accessory proteins. It also encodes four structural proteins—Spike (S), Nucleocapsid (N), Membrane (M), and Envelope (E) proteins [11] (Figure 1a)—which play important roles in the viral life cycle, host infection, and pathogenicity [12,13]. While the S, M, and E proteins mainly contribute to the outer shell structure of the virus particle, the N protein forms the viral capsid by packaging the viral RNA genome into a helical ribonucleoprotein (RNP) complex [13,14]. Thus, the N protein plays a pivotal role in protecting the viral genome by forming a nucleocapsid within the viral particle, making it less prone to mutation [15,16]. The N protein also participates in other processes such as viral genome transcription, replication, and immune regulation by interacting with viral and host proteins. The N protein is highly immunogenic, the most abundant viral protein during infection, and highly conserved among other coronaviruses. These specific features make the N protein a potential target for antiviral drug development and diagnosis, and hence an attractive subject for structural studies [12,17,18,19,20,21,22,23].

Numerous efforts have been made to understand the structure and function of coronaviruses’ N proteins. The SARS-CoV-2 N protein consists of 419 amino acids and comprises two structural domains: the N-terminal domain (N-NTD; residues 49–174) and the C-terminal domain (N-CTD; residues 247–364). These two domains are connected by a highly flexible linker region (residues 175–246), and at either end of these domains are intrinsically disordered regions, namely the N-arm (at the N-terminus; residues 1–48) and the C-tail (at the C-terminus; residues 365–419) [24] (Figure 1b). While the N protein interacts with RNA through the N-NTD, the N-CTD is involved in the self-dimerization of full-length N protein and RNA binding [25,26,27,28,29,30,31,32]. The flexible linker region (residues 175–246) has been shown to regulate the interaction of the N protein with RNA as well as with other viral and host proteins [33,34]. Though it is believed that the disordered C-tail plays a role in interaction with viral M protein and the packaging signal [22,35], the role of the N-arm remains unclear. Many structures of the N-NTD and N-CTD domains of the SARS-CoV-2 N protein have been solved separately [23,25,28,29,30,31,32,36,37,38,39,40,41,42,43,44]. Although the nucleocapsid (N) protein is a key component of the SARS-CoV-2 virus and an important target for therapeutic development, no high-resolution structure of the full-length N protein is currently available. Recently, structures of the full-length SARS-CoV-2 N protein were resolved by Cryo-EM at 6.00 Å and 4.57 Å resolutions as a dimer and monomer, respectively [45], but these were insufficient in obtaining detailed, precise information about the molecule. Thus, a high-resolution structure of the full-length SARS-CoV-2 N protein is still needed.

Escalating research on the N protein requires the isolation and purification of high-quality N protein in large quantities, which is generally achieved by overexpressing the recombinant protein in bacterial cells. Several groups have reported the purification of recombinant N protein from *E. coli* using affinity tags [31,46,47,48,49]. Although affinity tags simplify the protein purification process, retention or incomplete removal of the tags is not always ideal for downstream studies with the protein [50,51,52,53,54,55]. Furthermore, the method used for purification can also impact the quality and molecular properties of the N protein [56]. Therefore, developing a procedure to purify high-quality, full-length SARS-CoV-2 N protein without an affinity tag is valuable. Here, we report an affinity tag-free purification method for recombinant, full-length SARS-CoV-2 N protein in a large quantity and with high purity. Our purified N protein is highly soluble, homogeneous, free of nucleic acids, and suitable for biochemical, biophysical, and structural studies.

Although the main role of the SARS-CoV-2 N protein in the viral life cycle is to form the viral nucleoprotein complex (RNP) by interacting with genomic RNA and assembling the nucleoprotein complex, the detailed mechanism of this process is not well understood. Recent studies have also highlighted the involvement of the SARS-CoV-2 N protein in interactions with ssDNA and dsDNA. The ability of the SARS-CoV-2 N protein to unwind dsDNA as well as to anneal ssDNA may significantly interfere with host cell DNA replication, repair, and recombination processes. Additionally, the inhibition of SARS-CoV-2 replication by DNA aptamers shows promise for designing DNA-based inhibitors targeting the N protein [57,58,59,60]. To advance our understanding of nucleocapsid assembly formation and nucleic acid interactions, we aimed to crystallize the full-length N protein in complex with nucleic acids (ssDNA). Our crystallization efforts resulted in a high-resolution (1.55 Å) structure of the N-NTD with a small part of the N-arm in complex with ssDNA. The crystal packing of our structure suggests a possible role for the flexible N-arm in RNP assembly formation. Our structure showed that ssDNA binds to the N-NTD at the same RNA binding pocket, but the mode of interaction differs from that of RNA. Additionally, our structure revealed the amino acid residues important for ssDNA binding. As the inhibition of nucleocapsid assembly is a major pathway for antiviral development, our structure could be valuable for the structure-guided design of inhibitors.

## 2. Results and Discussion

### 2.1. N Protein Production

**Figure 2:**
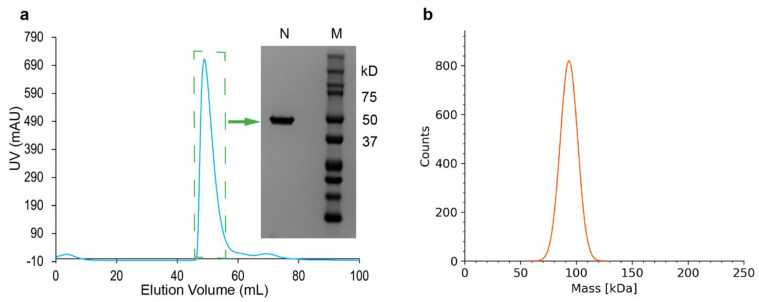
SARS-CoV-2 N protein purified by polyethyleneimine precipitation followed by SP Sepharose ion-exchange and finally size-exclusion chromatography. (**a**) FPLC size-exclusion chromatography and SDS-PAGE analysis of the purified N protein. The blue line represents the UV absorption spectrum of the eluted protein. The dashed green box highlights the absorption of the collected pure N protein. ‘N’ indicates the purified full-length N protein, while ‘M’ represents the protein marker. (**b**) Mass photometry analysis revealed that the N protein appeared as a monodisperse dimer (~93 kD) in-solution.

Considering the high demand for high-quality full-length SARS-CoV-2 N protein, we developed an *E. coli* overexpression vector for the SARS-CoV-2 N gene without any affinity tag. We used a polyethyleneimine (PEI) precipitation method to separate nucleic acids from proteins. The supernatant was treated with ion-exchange chromatography (SP Sepharose) followed by size-exclusion chromatography (Superdex-75), which allowed us to purify full-length N protein. Size-exclusion chromatography and SDS-PAGE analysis (Figure 2a) revealed the eluted protein was a single, major species with high purity. UV absorption analysis confirmed the absence of nucleic acid contamination in the purified N protein. As reported by other groups [16,22], our mass photometry (MP) analysis showed that the N protein remained as a homogeneous dimer at a concentration of 50 to 100 nM (Figure 2b). Overall, our purified N protein was highly soluble, homogeneous, nucleic acid-free, and suitable for biochemical, biophysical, and structural studies.

### 2.2. Crystal Formation and Structure of N-NTD with Part of Flexible N-Arm

We aimed to capture the oligomeric structure of the full-length N protein in complex with nucleic acids. As reported by Zhao et al. [61], dimeric N protein’s tendency to form higher-order oligomers depends on the nucleic acid length and shows a strong binding affinity with 10 nt poly-T (T-10) and 20 nt poly-T (T-20), forming higher-order oligomers. Accordingly, we attempted to crystallize the full-length N protein in complex with T-10 (10 nt T) and T-20 (20 nt T) ssDNA. After approximately a year, crystals appeared in a single condition (F4 condition from PEG/pH screen, Hampton Research) at 4 °C, in a well with N protein and T-20 ssDNA (Supplementary Figure S1a). A cryoprotected crystal diffracted at high resolution (Supplementary Figure S1b). The crystal belonged to the I 41 space group, with unit cell parameters (a = b = 90.89 Å, c = 36.34 Å, α = β = γ = 90°) (Table 1). Cell content analysis using the CCP4 program suit suggested that the unit cell volume was insufficient to accommodate the full-length N protein but could contain half of it.

Molecular replacement using the available structural coordinates of N-NTD (PDB ID:7N0R) and N-CTD (PDB ID:6ZCO) together (for the full-length protein) or using only N-CTD (for the C-terminal domain) failed to produce a solution. However, molecular replacement with only N-NTD (for the N-terminal domain) successfully provided a solution, resulting in a single-protein molecule per asymmetric unit and final structure determination at 1.55 Å resolution. The crystallization of only the N-terminal part of the full-length N protein, after an extended period, led us to hypothesize that the full-length N protein might have been digested in the crystallization condition, with only the N-terminal part crystallizing under the given condition.

To confirm this, we collected the residual crystallization sample (RS) after crystal formation in the N+T-20 crystallization well, and the crystallization sample (CS) from the N+T-10 crystallization well without crystal formation, and examined them by SDS-PAGE. The SDS-PAGE analysis confirmed that the full-length protein was indeed digested under the crystallization condition, producing a significant fragment with an approximate molecular weight of 20 kD (Supplementary Figure S1d,e). Supporting previous reports, our findings indicate that the crystallization of full-length N protein is challenging due to its intrinsic flexibility and protease sensitivity [16,23,62,63].

**Figure 3:**
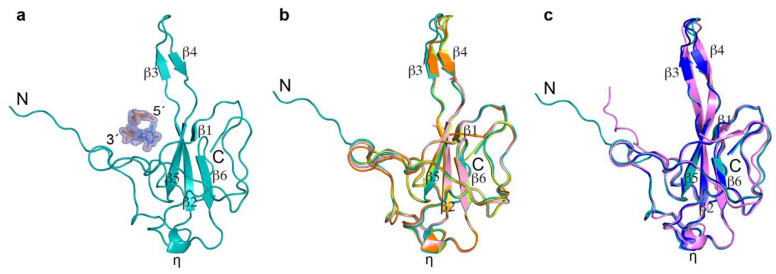
Comparisons of coronavirus N protein structures. (**a**) Our N protein structure (digested full-length N) showing N-NTD with part of the flexible N-arm (PDB ID:9CJ6; teal color, cartoon) in complex with ssDNA (blue, stick). A 2Fo–Fc electron density map contoured at 1σ is shown in light blue around the ssDNA. (**b**) Superimposition of our structure with other published SARS-CoV-2 N-NTD structures (PDB ID:7XWZ; light pink color, PDB ID:7CDZ; orange color, PDB ID:7N0R; limon color). (**c**) Superimposition of our structure with closely related SARS-CoV N-NTD (PDB ID: 2OFZ; navy blue color) and MERS-CoV- N-NTD (PDB ID: 4UD1; purple color) structures. N and C indicate the N- and C-terminal ends of the protein, respectively. The 5′ and 3′ represent the 5′ end and 3′ end of ssDNA. ß and η represent the ß-strand and short 3_10_ helix, respectively.

Although we could not determine the exact length of the crystallized fragment of the N protein, we were able to build a structural model based on the electron density observed after phase determination via molecular replacement. Like previously reported N-NTD structures, strong electron density was observed for the N-NTD structured region (residues 49–173) along with a part of the flexible N-arm (residues 39–48), allowing us to confidently build the structural model. No electron density was observed for the rest of the protein, and it was therefore not modeled in the final crystal structure. Overall, we solved the crystal structure of the N protein from residues 39 to 173, covering part of the flexible N-arm and the entire N-NTD (Supplementary Figure S1c,f and Figure 3a).

We also observed strong electron density for a nucleotide along with additionally strong density for two adjoining phosphates, but weak density for the corresponding base. Since our crystallization sample contained 20 nt T, the electron density observed here could represent a stable portion of the poly-T interacting with N-NTD, while the remaining poly-T remains flexible and invisible. Therefore, we modeled the trinucleotide as TTT (Supplementary Figure S1c and Figure 3a).

### 2.3. Comparison with Published N-NTD Structures

Since the COVID-19 pandemic, several structures of SARS-CoV-2 N-NTD have been published (Supplementary Table S1): some in complex with dsRNA or ssRNA [28,30], some with antibodies or proteins [37,38,42,44], some with small molecules, and some in the apo form [23,25,29,30,44]. Among all the crystal structures deposited in the PDB, the apo N-NTD has been crystallized as a trimer (PDB ID: 6VYO, 6WKP, 7UW3, 6M3M, 7CDZ, 7XX1, and 7VBD); N-NTD in complex with dsRNA has been crystallized as a dimer (PDB ID: 7XWZ); and N-NTD in complex with antibodies or proteins has been crystallized as a monomer (PDB ID: 7STR, 7STS, 7SUE, 7N3C, 7N3D, 7CR5, 7N0R, 7R98, and 7VNU) in the asymmetric unit. Our N-NTD in complex with ssDNA crystallized as a monomer in the asymmetric unit (Figure 3a) and maintained good topological agreement with other published crystal structures of SARS-CoV-2 N-NTD (Figure 3b), as well as with the closely related SARS-CoV (PDB ID: 2OFZ) and MERS-CoV N-NTD (PDB ID: 4UD1) structures (Figure 3c).

The N-NTD resembles a right-handed fist, containing a core of four-stranded antiparallel ß-sheets (ß1-ß5-ß2-ß6), which is sandwiched between two loops, a short 3_10_ helix (η), and a protruding ß-hairpin (ß3 and ß4) between ß2 and ß5 (Figure 3a–c). As previously reported, the protruding ß-hairpin (ß3 and ß4) region is flexible, exhibiting a range of dynamic conformations across different structures. Most previous N-NTD structures were determined using the stable N-NTD construct spanning residues 48 to174. However, in our structure (a digested full-length N), in addition to the stable N-NTD, we determined the structure of a small part of the flexible N-arm at high resolution. The N-arm extends outward from the N-NTD core, like what has been observed in the MERS-CoV N-NTD structure (Figure 3c) [64].

### 2.4. Putative Role of N-Arm in Nucleocapsid Assembly

In the viral life cycle, the primary role of the N protein is to bind viral genomic RNA to form the viral nucleoprotein complex (RNP) and assemble this complex within the virus particle. Previous studies have indicated that the assembly of the RNP complex involves multiple regions of the N protein that mediate protein–RNA and protein–protein interactions [17,22,65,66,67]. However, the mechanism of RNP complex assembly formation remains elusive.

It is known that intrinsically disordered proteins (IDPs) or intrinsically disordered regions (IDRs) of a protein lack a defined structure yet play important roles in biological processes, especially in macromolecular interactions [68,69,70,71]. Due to its intrinsically disordered nature, the structural information and biological role of the N-arm of the SARS-CoV-2 N protein are still unclear.

**Figure 4:**
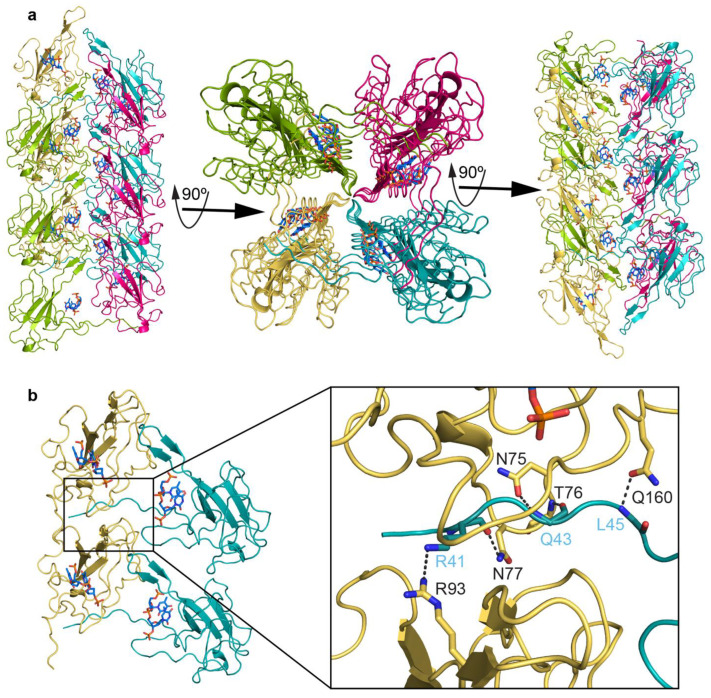
Crystal packaging of digested N protein. (**a**) In the crystal, four neighboring protein molecules are colored as teal, hot pink, split pea, and yellow-orange, showing crystal packing at different 90° angles. (**b**) The N-arm extends to neighboring molecules and is anchored between two molecules through hydrogen bonding. Gray dashed lines represent the hydrogen bonds between amino acids.

Our crystal structure revealed that the N-NTD, along with part of the flexible N-arm, arranges in a specific pattern where the N-arm extends to neighboring molecules and anchors between two molecules through significant hydrogen bonding (Figure 4). Specifically, the mainchain amine of L45 forms a direct hydrogen bond with the sidechain carbonyl of Q160 in loop 2. The mainchain amine and carbonyl of Q43 form hydrogen bonds with the sidechain carbonyl of N75 and the mainchain amine of T76, respectively, in loop 1. The mainchain carbonyl of R41 forms a hydrogen bond with the sidechain amine of N77 in loop 1. Additionally, the sidechain nitrogen of R41 forms a hydrogen bond with the sidechain NH_2_ of R93 (near ß3) of another neighboring molecule. The crystal packing of our structure suggested a possible role for the flexible N-arm in RNP assembly formation. However, further experiments are required to support this hypothesis.

### 2.5. ssDNA Interacts at the RNA Binding Pocket of N-NTD with Different Interaction Modes

Despite the main role of the N protein in packaging the viral RNA genome, new findings highlight several biological processes that require the interaction of the N protein with ssDNA or dsDNA [57,58,59]. Previous computational and structural studies have provided significant insights into how the N protein interacts with dsRNA and ssRNA [28,30]. However, structural information on how the N protein interacts with dsDNA or ssDNA is lacking. To address this gap, we aimed to crystallize poly-T (T-10 and T-20) in complex with the N protein.

In our structure, we were able to model a small segment (TTT) of the T-20 ssDNA based on well-defined electron density. The absence of electron density for other parts of the ssDNA may have been due to the dynamic nature of ssDNA or possible degradation under the crystallization condition [72,73,74]. As we were uncertain about which segment of the TTT nucleotide stretched stably bound to the N protein and got crystallized, we numbered the T with well-defined electron density as 0, with −1 and +1 indicating the positions toward the 5′ and 3′ ends, respectively. Nevertheless, our structure provides valuable insights into the interaction between the N protein and ssDNA.

**Figure 5:**
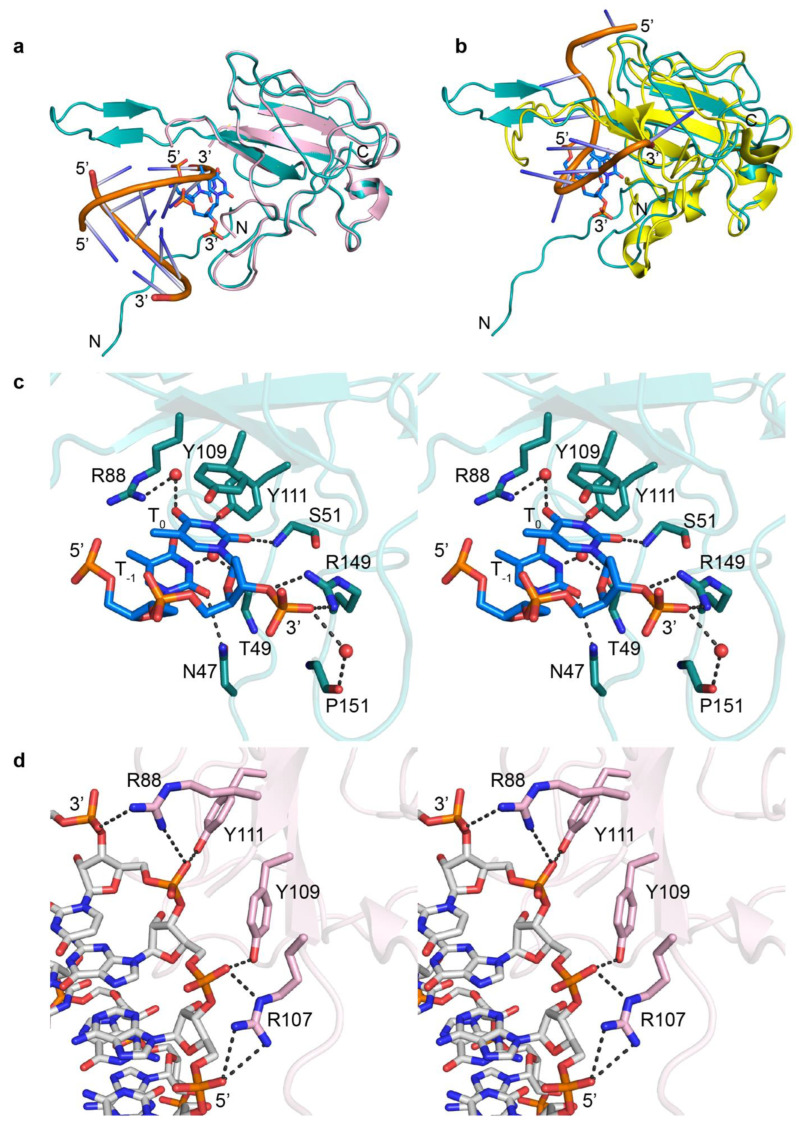
Interactions of N protein with nucleic acids. (**a**) Superimposition of our structure (teal, PDB ID: 9CJ6) with the crystal structure of the N-NTD complex with dsRNA (light pink, PDB ID: 7XWZ). (**b**) Superimposition of our structure (teal, PDB ID: 9CJ6) with the NMR structure of the N-NTD complex with ssRNA (yellow, PDB ID: 7ACT). RNA is shown as a cartoon and ssDNA as a stick. ssDNA binds to N-NTD at the RNA binding pocket. (**c**) Stereo-view of detailed interaction of ssDNA with N-NTD (teal, our structure). (**d**) Stereo view of the detailed interaction of dsRNA with N-NTD (light pink, PDB ID: 7XWZ). ssDNA and RNA are represented as sticks, and water is represented as the red sphere. Gray dashed lines represent the hydrogen bonds. The nucleic acid bases of ssDNA interact with protein through direct and water-mediated hydrogen bonds. The RNA interacts with protein through hydrogen bonds in the phosphate backbone.

Superimposing our structure with previous structures of N-NTD in complex with dsRNA and ssRNA (PDB ID: 7XWZ and 7ACT) showed that ssDNA binds to the N-NTD at the same RNA binding pocket, but in a different mode (Figure 5a,b). The Watson–Crick face of ssDNA interacts with the protein through direct and water-mediated hydrogen bonds as well as π–π stacking (Figure 5c). The Watson–Crick face of T_0_ forms two direct and one water-mediated interaction with the protein. The 2-carbonyl group forms a hydrogen bond with the mainchain amino proton of S51, the N3 atom forms a hydrogen bond with the sidechain –OH group of Y111, and the 4-carbonyl group forms a water-mediated hydrogen bond with the guanidino group of R88. T_0_ also stacks with Y109 through π–π stacking. Additionally, the deoxyribose O3ʹ of T_0_ forms a direct hydrogen bond with the guanidino group of R149 (Figure 5c and Supplementary Figure S3). The Watson–Crick face of T_−1_ interacts with the protein through one direct and one water-mediated hydrogen bond. The 2-carbonyl group forms a hydrogen bond with the sidechain amino group of N47, and the N3 atom forms a water-mediated hydrogen bond with the mainchain carbonyl group of T49. Additionally, the 5′-phosphate group of T_+1_ is supported by one direct hydrogen bond with the guanidino group of R149 and one water-mediated hydrogen bond with the mainchain carbonyl group of P151 (Figure 5c and Supplementary Figure S3). In contrast, RNA interacts with the N protein through its phosphate backbone (Figure 5d) [28,30]. Residues R88, Y111, Y109, and R149 are involved in interactions with both DNA and RNA.

Recent studies have highlighted the use of oligonucleotides as potential inhibitors of protein function [75]. A designed ssDNA aptamer has shown promise as an antiviral therapy against COVID-19 by disrupting the liquid–liquid phase separation (LLPS) mediated by the N protein [59]. Our structure, along with previous structures, will aid in designing more effective nucleic acid-based therapeutics against SARS-CoV-2.

## 3. Materials and Methods

### 3.1. Plasmid Generation and Protein Overexpression and Purification

The protein expression vector was generated by cloning the SARS-CoV-2 N gene (*Gene ID: 43740575*) into the pET-11a vector (GenScript) using cloning sites 5′ NdeI and 3′ BamHI, without any affinity tag. The *pET-11a* expression vector carrying the SARS-CoV-2 N gene was transformed into BL21 (DE3) cells (Invitrogen). Cells were grown in LB medium at 37 °C until they reached an optical density of 0.5–0.6 at 600 nm. The temperature was then reduced to 17 °C, and protein overexpression was induced by adding 0.2 mM IPTG at an optical density of 0.6–0.8. Cells were further grown overnight at 17 °C. All protein purification steps were performed at 4 °C unless otherwise specified.

*E. coli* cells from a 1 L culture were harvested by centrifugation and resuspended in 25 mL of lysis buffer (20 mM Tris-HCl, pH 7.5, 200 mM NaCl, 1 mM DTT, 5% glycerol) with protease inhibitors (Roche, Basel, Switzerland). The suspended cells were disrupted by sonication, and cell debris was removed by centrifugation at 12,000 rpm for 45 min.

The supernatant was treated with a gradual addition of 5% polyethyleneimine to reach a final concentration of 0.5% polyethyleneimine in solution and stirred for 1 h to allow maximum precipitation. The precipitate was separated by centrifugation at 10,000 rpm for 10 min, and the supernatant containing the N protein was retained.

The supernatant was added to 6 mL of SP Sepharose beads (pre-equilibrated with lysis buffer) and agitated overnight. The N protein bound to the SP Sepharose beads was passed through a column under gravity. After washing the beads with 40 mL of lysis buffer, the protein was eluted with 10 mL of elution buffer in fractions E1–E6 (lysis buffer with varying salt concentrations: E1 = 0.3 M, E2 = 0.4 M, E3 = 0.5 M, E4 = 0.6 M, E5 = 0.8 M, and E6 = 1 M NaCl). The N protein was completely eluted in fractions E2 (0.4 M NaCl) and E3 (0.5 M NaCl), as confirmed by SDS-PAGE electrophoresis (Supplementary Figure S2a).

The eluted protein was further purified using a Superdex 75 size-exclusion column (GE Healthcare Life Sciences) with FPLC buffer (20 mM Tris-HCl, pH 7.5, 100 mM NaCl, 1 mM DTT, 1% glycerol) on an AKTA FPLC system (Supplementary Figure S2b). The purity and concentration of the proteins were measured by gel electrophoresis and UV spectroscopy.

### 3.2. Mass Photometry

Mass photometry experiments were performed using a TwoMP mass photometer (Refeyn Ltd., Oxford, UK). High-precision cover glasses (24 × 50 mm, thickness No. 1.5H, Paul Marienfeld GmbH and Co KG, Lauda-Königshofen, Germany) and six-well pre-cut CultureWell gaskets (Grace Bio-Labs, Bend, OR, USA) were used. Coverslips were rinsed sequentially with deionized water and isopropyl alcohol (IPA) in the following order, water–IPA–water–IPA–water, and then dried with a stream of ultrapure nitrogen. A drop of Immersol 518F immersion oil (Carl Zeiss Jena, Oberkochen, Germany) was placed on the microscope lens before placing the coverslips on the mass photometer stage.

Protein samples were prepared at concentrations of 50 nM and 100 nM in sample buffer (20 mM Tris-HCl, pH 7.5, 100 mM NaCl, 1 mM DTT). The instrument was calibrated with a mix of beta-amylase from sweet potato and thyroglobulin from bovine thyroid (Sigma-Aldrich), both dissolved in sample buffer. Autofocusing was performed in buffer-free mode, and 15 μL of protein solution was placed in a well on the coverslip. Movies were recorded for 60 s using the regular view setting in AcquireMP (version 2023 R1.1) software. The experiments were performed in triplicate with freshly prepared samples. Data analysis was conducted in histogram mode with the manual selection of observed peaks, followed by Gaussian fitting using DiscoverMP software (version 2023 R1.2).

### 3.3. Crystal Growth and Data Collection

We aimed to crystallize the full-length SARS-CoV-2 N protein in complex with ssDNA. For this purpose, we used two different ssDNAs, T-10 (TTTTTTTTTT) and T-20 (TTTTTTTTTTTTTTTTTTTT), as reported by Zhao et al. [61]. T-10 and T-20, at 10% molar excess, were each mixed separately with purified SARS-CoV-2 N protein at a concentration of 30 μM. The mixtures were then concentrated to approximately 900 μM protein concentration using Amicon Ultra-4 (Merck Millipore). Crystallization screening was performed using various commercially available crystallization screens and the sitting drop vapor diffusion method at 4 °C and 20 °C. Crystal drops were prepared by mixing 0.3 μL of the sample with 0.3 μL of reservoir solution in a 2-well sitting drop crystallization plate (Molecular Dimension) using a Mosquito Crystal robot (TTP Labtech). Crystals appeared after approximately one year under a condition containing 0.2 M HEPES (pH 7.4) and 20% *w/v* PEG 4000 (F4 condition from PEG/pH screen, Hampton Research). Crystals were cryoprotected using the reservoir solution supplemented with 15% *v/v* glycerol and flash-frozen in liquid nitrogen. X-ray diffraction data were collected at the Southeast Regional Collaborative Access Team (SER-CAT) 22-ID beam line at the Advanced Photon Source, Argon National Laboratory.

### 3.4. Structural Determination and Analysis

The collected diffraction data were indexed, integrated, and scaled using the HKL2000 program [76]. The crystals belong to the space group I 41, with unit cell parameters (a = b = 90.89 Å, c = 36.34 Å, α = β = γ = 90°) (Table 1). Cell content analysis was performed using Mathew’s coefficient [77] in the CCP4 program suit. The structure was solved at 1.55 Å resolution by molecular replacement using the program Phaser [78] and the N-NTD structure (PDB ID:7N0R) as the search model. Model building of the protein and bound DNA was manually performed using the program Coot [79]. The structural model was refined with Phenix refinement [80,81], and the final model was validated using the PDB validation tool (https://validate.wwpdb.org) and MolProbity [82]. Structural figures were created using the software PyMOL. Data collection statistics and refinement parameters of the crystal structure are provided in Table 1.

## 4. Conclusions

In this study, we developed an optimized protocol for the affinity tag-free purification of the SARS-CoV-2 N protein with a high yield. Our purification method involved polyethyleneimine precipitation, SP Sepharose chromatography, and size-exclusion chromatography, resulting in the isolation of highly pure N protein. The purified N protein was highly soluble, homogeneous, nucleic acid-free, and suitable for structural studies. Mass photometry analysis confirmed that the purified N protein dimerized in solution, consistent with previous reports. Our purification method provides a reliable source of N protein for the further exploration of its biochemical and biophysical properties, particularly its interactions with nucleic acids.

Despite our efforts to crystallize the full-length N protein in complex with ssDNA, we encountered the inherent challenges posed by its flexibility and protease sensitivity, leading to the crystallization of only the N-terminal domain (N-NTD). This result also underscores the possibility of the spontaneous cleavage of the SARS-CoV-2 N protein. As previously observed, under certain conditions, the N-terminal sides of Ser and Thr residues are prone to spontaneous cleavage [83,84]. Notably, the flexible linker region of the SARS-CoV-2 N protein contains several Ser and Thr residues, making it highly susceptible to such cleavage. Our findings provide an important insight for researchers, emphasizing the need to carefully assess the stability of full-length N protein during biochemical and biophysical experiments, as well as during crystallization trials.

Our structural analysis revealed that the N-NTD, along with the flexible N-arm, formed a specific arrangement that facilitated higher-order oligomerization and suggests a potential contribution to the assembly of the viral ribonucleoprotein complex (RNP). Notably, the crystallized N-NTD displayed a monomeric form in complex with a trinucleotide, suggesting a distinct mode of interaction with ssDNA compared to RNA. These structural insights, combined with previous findings, expand our understanding of the N protein’s role in viral assembly and its potential as a target for nucleic acid-based therapeutics. The unique binding mode of ssDNA observed in our structure highlights key residues involved in this interaction, providing a foundation for future therapeutic development. Given the challenges of crystallizing the full-length N protein, further experiments are required to fully elucidate its role in RNP assembly and its interactions with different nucleic acids. These findings open new avenues for designing inhibitors targeting the N protein, with the potential to disrupt SARS-CoV-2 replication and pathogenesis.

## Figures and Tables

**Table 1:** Data collection and refinement statistics.

*Data Collection*	
Space group	I 41
Cell dimensions a, b, c (Å) α, β, γ (°)	90.89, 90.89, 36.34 90.00, 90.00, 90.00
Resolution (Å)	50.00–1.55 (1.61–1.55) *
R_merge_ (%)	7.0 (65.3)
R_pim_ (%)	1.9 (18.6)
I/σI	34.42 (2.94)
CC1/2	0.998 (0.945)
Completeness (%)	99.8 (97.0)
Redundancy	12.9 (11.6)
*Refinement*	
Resolution (Å)	33.74–1.55 (1.61–1.55)
No. of reflections	20,986 (1666)
R_work_/R_free_ (%)	17.80/20.20
No. of atoms	2193
Protein	1972
DNA	44
Water	177
B-factor	
Average B-factors (Å^2^)	21.0
Protein/DNA	19.2
Water	30.1
Ramachandran plot (%)	
Favored regions	98.5
Allowed regions	1.5
Outliers	0
r.m.s deviations	
Bond lengths (Å)	0.009
Bond angles (°)	1.26

* Values in parentheses are for highest-resolution shell.

## Data Availability

Atomic coordinates and structural factors were deposited in the Protein Data Bank with accession code 9CJ6 (https://www.rcsb.org/structure/9CJ6). Data are available from A.M. and H.M upon request (e-mail: atanu.maiti@nih.gov and hiroshi.matsuo@nih.gov).

## Supplementary Materials

The following supporting information can be downloaded at: https://www.mdpi.com/article/10.3390/biom14121538/s1. Figure S1: Crystallization of SARS-CoV-2 N protein with Poly-T (T-20) ssDNA; Figure S2: SDS-PAGE analysis of SARS-CoV-2 N protein expression and purification; Figure S3: Summary of the interactions between SARS-CoV-2 N-NTD and nucleotides TTT; Table S1: Structures of SARS-CoV-2 N-NTD reported in Protein Data Bank (PDB).
