# Supplementary material for: Affinity Tag-Free Purification of SARS-CoV-2 N Protein and Its Crystal Structure in Complex with ssDNA
Source: Biomolecules. 2024 Nov 30;14(12):1538. doi: 10.3390/biom14121538 (PMC11673995; doi:10.3390/biom14121538)
Supplement: Supplementary file 1 [file biomolecules-14-01538-s001.zip › biomolecules-3244999-supplementary.pdf]

Supplementary Materials:

Affinity tag free purification of SARS-CoV-2 N protein and its crystal structure in complex with ssDNA.

Atanu Maiti[1*] and Hiroshi Matsuo[1*]

[1]Cancer Innovation Laboratory, Frederick National Laboratory for Cancer Research, Frederick, MD, USA.

* To whom correspondence should be addressed.
Atanu Maiti, Ph.D.
Tel: +1 (301) 846-6826
e-mail: atanu.maiti@nih.gov

Hiroshi Matsuo, Ph.D.
Tel: +1 (301) 228-4375
e-mail: hiroshi.matsuo@nih.gov

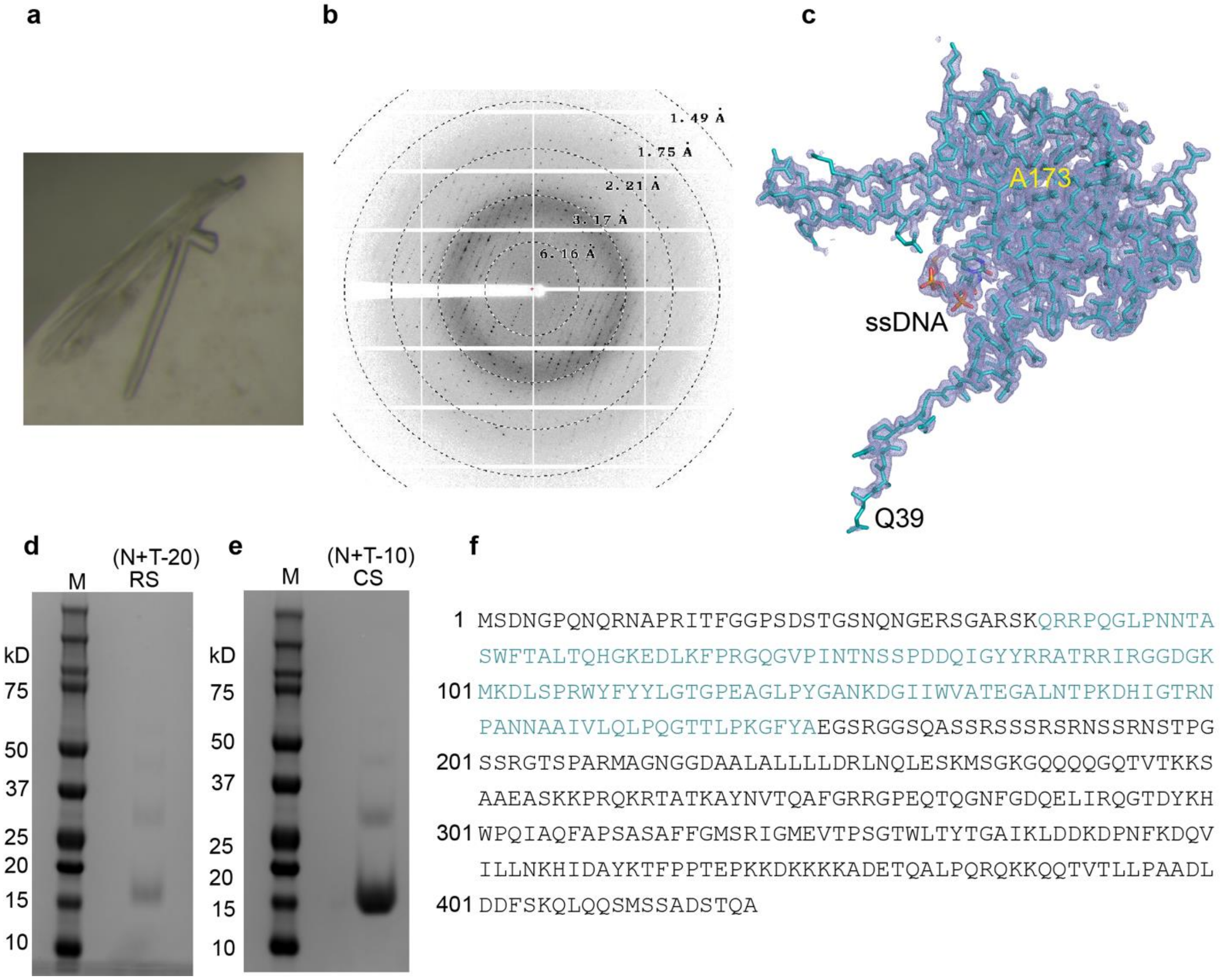

Supplementary Figure S1: Crystallization of SARS-CoV-2 N protein with Poly-T (T-20) ssDNA. a) Crystal appeared in a condition after approximately a year. b) Cryoprotected crystal diffracted at high resolution. c) The crystal appeared in the I 41 space group with unit cell parameter (a= b=90.89Å, c=36.34Å, α=β=γ=90°). The cell content analysis suggested that the unit cell volume can accommodate half of the full-length N protein. Molecular replacement with only N-NTD (PDB ID:7N0R) successfully provided a solution, resulting a single protein molecule per asymmetric unit. A 2Fo–Fc electron density map contoured at 1σ is shown in light blue around the protein and ssDNA. d & e) The SDS-PAGE analysis of residual crystallization sample (RS) after crystal formation in the N+T-20 crystallization well, and the crystallization sample (CS) from the N+T-10 crystallization well without crystal formation, confirmed that the full-length N protein was digested under the crystallization condition. c & f) We modeled residues Q39 to A173 (blue sequence) of full-length N (black and blue sequence) protein in complex with ssDNA.

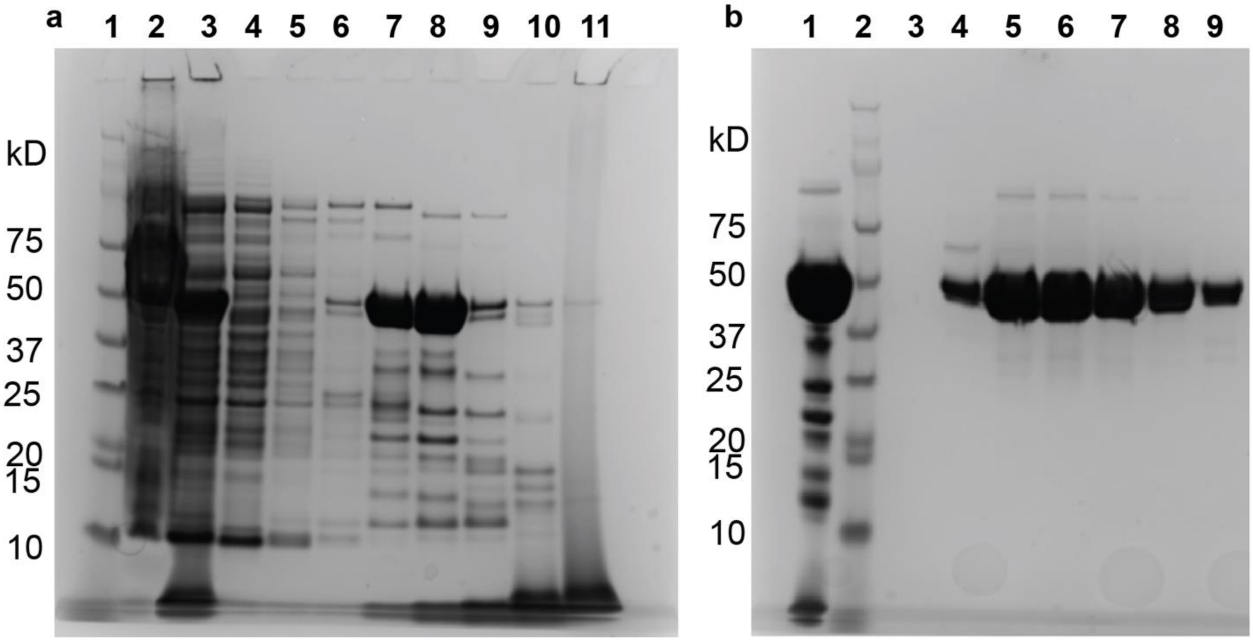

Supplementary Figure S2: SDS-PAGE analysis of SARS-CoV-2 N protein expression and purification. a) Lane-1: Precision Plus Protein Standards (Bio-Rad); lane-2: Supernatant after sonication; lane-3: Supernatant after polyethyleneimine precipitation; lane-4: SP-column flow through; lane-5: SP-column wash; lane-6: Elution 1 (E1=0.3M NaCl); lane-7: Elution 2 (E2=0.4m NaCl); lane-8: Elution 3 (E3=0.5M NaCl); lane-9: Elution 4 (E4=0.6M NaCl); lane-10: Elution 5 (E5=0.8M NaCl); lane-11: Elution 6 (E6=1M NaCl). b) SDS-PAGE of Superdex-75 size exclusion chromatography. Lane-1: injected sample, lane-2: Precision Plus Protein Standards (Bio-Rad), Lane-3-9: FPLC fractions 14-20.

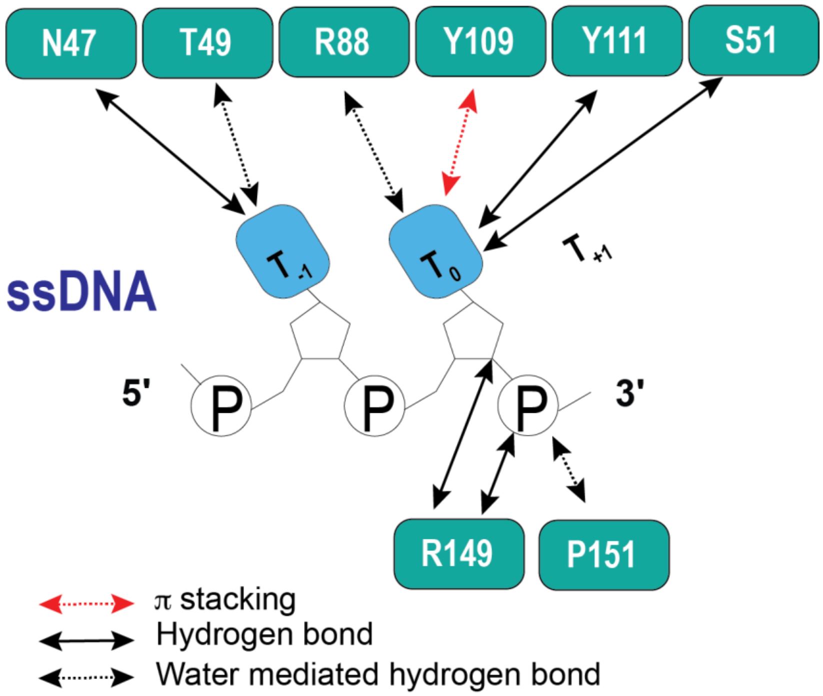

Supplementary Figure S3: Summary of the interactions between SARS-CoV-2 N-NTD and nucleotides TTT. Rectangular boxes (teal color) represent the amino acid residues involved in the interactions.

Supplementary Table S1: Structures of SARS-CoV-2 N-NTD reported in Protein Data Bank (PDB)

Structures of SARS-CoV-2 N-NTD reported in Protein Data Bank (PDB)						
	PDB ID	Method and Resolution	Molecule / length of the N-NTD domain	Apo /Complex	Oligomeric state	References
1.	7XX1	X-Ray 1.90 Å	SARS-CoV-2 N-NTD, aa 49-173	N-NTD Apo	N-NTD Tetramer	Antiviral drug design based on structural insights into the N-terminal domain and C-terminal domain of the SARS-CoV-2 nucleocapsid protein. Xiaodong Luan , Xinming Li , Yufan

						Li , Gengchen Su, Wanchao Yin, Yi Jiang , Ning Xu , Feng Wang , Wang Cheng , Ye Jin , Leike Zhang , H Eric Xu , Yi Xue , Shuyang Zhang , (2022) Sci Bull (Beijing) 67: 2327-2335
2	7XWZ	X-Ray 2.25 Å	SARS-CoV-2 N-NTD, aa 48-171	N-NTD-dsRNA complex, dsRNA sequence (5′CACUGAC-3′ and 5′GUCAGUG-3′)	N-NTD Dimer with two dsRNA	Antiviral drug design based on structural insights into the N-terminal domain and C-terminal domain of the SARS-CoV-2 nucleocapsid protein. Xiaodong Luan , Xinming Li , Yufan Li , Gengchen Su, Wanchao Yin, Yi Jiang , Ning Xu , Feng Wang , Wang Cheng , Ye Jin , Leike Zhang , H Eric Xu , Yi Xue , Shuyang Zhang , (2022) Sci Bull (Beijing) 67: 2327-2335
3.	7CDZ	X-Ray 1.80 Å	SARS-CoV-2 N-NTD, aa 47-174,	N-NTD Apo	N-NTD Tetramer	Structures of the SARS-CoV-2 nucleocapsid and their perspectives for drug design. Ya Peng , Ning Du , Yuqing Lei , Sonam Dorje , Jianxun

						Qi , Tingrong Luo , George F Gao , Hao Song , (2020) EMBO J 39: e105938-e105938
4.	6M3M	X-Ray 2.70 Å	SARS-CoV-2 N-NTD, aa 48-173	N-NTD Apo	N-NTD Tetramer	Crystal structure of SARS-CoV-2 nucleocapsid protein RNA binding domain reveals potential unique drug targeting sites.<br><br>Sisi Kang , Mei Yang , Zhongsi Hong , Liping Zhang, Zhaoxia Huang , Xiaoxue Chen , Suhua He , Ziliang Zhou , Zhechong Zhou , Qiuyue Chen , Yan Yan , Changsheng Zhang , Hong Shan , Shoudeng Chen , (2020) Acta Pharm Sin B 10: 1228-1238

5.	7CR5	X-Ray 2.08 Å	SARS-CoV-2 N-NTD, aa 48-172	N-NTD-Antibody nCoV396Fab complex	N-NTD monomer	A SARS-CoV-2 antibody curbs viral nucleocapsid protein-induced complement hyperactivation.<br>Sisi Kang , Mei Yang , Suhua He , Yueming Wang , Xiaoxue Chen , Yao-Qing Chen , Zhongsi Hong , Jing Liu , Guanmin Jiang , Qiuyue Chen , Ziliang Zhou , Zhechong Zhou , Zhaoxia Huang , Xi Huang , Huanhuan He , Weihong Zheng , Hua-Xin Liao , Fei Xiao , Hong Shan , Shoudeng Chen ,<br>(2021) Nat Commun 12: 2697-2697
6.	7N0R	X-Ray 1.42 Å	SARS-CoV-2 N-NTD, aa 49-174	N-NTD-single-domain Antibody-C2 complex	N-NTD monomer	Structural Basis for SARS-CoV-2 Nucleocapsid Protein Recognition by Single-Domain Antibodies.<br>Qiaozhen Ye , Shan Lu , Kevin D Corbett ,<br>(2021) Front Immunol 12: 719037-719037

7.	7R98	X-Ray 2.51 Å	SARS-CoV-2 N-NTD, aa 49-174	N-NTD-single-domain Antibody-E2 complex	N-NTD monomer	Structural Basis for SARS-CoV-2 Nucleocapsid Protein Recognition by Single-Domain Antibodies.<br>Qiaozhen Ye , Shan Lu , Kevin D Corbett ,<br>(2021) Front Immunol 12: 719037-719037
8.	7UW3	X-Ray 1.70 Å	SARS-CoV-2 N-NTD, aa 48-174	N-NTD apo	N-NTD tetramer	Atomic-Resolution Structure of SARS-CoV-2 Nucleocapsid Protein N-Terminal Domain.<br>Sucharita Sarkar , Brent Runge , Ryan W Russell , Kumar Tekwani Movellan , Daniel Calero , Somayeh Zeinalilathori , Caitlin M Quinn , Manman Lu , Guillermo Calero , Angela M Gronenborn , Tatyana Polenova ,<br>(2022) J Am Chem Soc 144: 10543-10555

9.	7WZO	X-Ray 2.64 Å	SARS-CoV-2 N-NTD, aa 49-173	N-NTD-Ubl1 complex	N-NTD monomer	Structural insights into ribonucleoprotein dissociation by nucleocapsid protein interacting with non-structural protein 3 in SARS-CoV-2<br>Xincheng Ni , Yinze Han , Renjie Zhou , Yanmei Zhou , Jian Lei<br>(2023) Commun Biol 6: 193-193
10.	7VNU	X-Ray 1.95 Å	SARS-CoV-2 N-NTD, aa 47-174	N-NTD Apo	N-NTD Tetramer	Structural insights into ribonucleoprotein dissociation by nucleocapsid protein interacting with non-structural protein 3 in SARS-CoV-2<br>Xincheng Ni , Yinze Han , Renjie Zhou , Yanmei Zhou , Jian Lei<br>(2023) Commun Biol 6: 193-193

11.	6VYO	X-Ray 1.70 Å	SARS-CoV-2 N-NTD, aa 50-173	N-NTD Apo	N-NTD Tetramer	Epitopes recognition of SARS-CoV-2 nucleocapsid RNA binding domain by human monoclonal antibodies<br><br>Youngchang Kim, Natalia Maltseva, Christine Tesar , Robert Jedrzejczak , Michael Endres , Heng Ma , Haley L Dugan , Christopher T Stamper , Changsoo Chang , Lei Li , Siriruk Changrob , Nai-Ying Zheng , Min Huang , Arvind Ramanathan , Patrick Wilson , Karolina Michalska , Andrzej Joachimiak ,<br><br>(2024) iScience 27(2):108976.

12.	6WKP	X-Ray 2.67 Å	SARS-CoV-2 N-NTD, aa 50-173	N-NTD Apo	N-NTD Tetramer	Epitopes recognition of SARS-CoV-2 nucleocapsid RNA binding domain by human monoclonal antibodies<br><br>Youngchang Kim, Natalia Maltseva, Christine Tesar , Robert Jedrzejczak , Michael Endres , Heng Ma , Haley L Dugan , Christopher T Stamper , Changsoo Chang , Lei Li , Siriruk Changrob , Nai-Ying Zheng , Min Huang , Arvind Ramanathan , Patrick Wilson , Karolina Michalska , Andrzej Joachimiak ,<br><br>(2024) iScience 27(2):108976.

13.	7STS	X-Ray 2.16 Å	SARS-CoV-2 N-NTD, aa 49-173	N-NTD-Human Fab S24-1379 complex	N-NTD monomer	Epitopes recognition of SARS-CoV-2 nucleocapsid RNA binding domain by human monoclonal antibodies<br><br>Youngchang Kim, Natalia Maltseva, Christine Tesar , Robert Jedrzejczak , Michael Endres , Heng Ma , Haley L Dugan , Christopher T Stamper , Changsoo Chang , Lei Li , Siriruk Changrob , Nai-Ying Zheng , Min Huang , Arvind Ramanathan , Patrick Wilson , Karolina Michalska , Andrzej Joachimiak ,<br><br>(2024) iScience 27(2):108976.

14.	7SUE	X-Ray 2.90 Å	SARS-CoV-2 N-NTD, aa 47-172	N-NTD-Human Fab S24-188 complex	N-NTD monomer	Epitopes recognition of SARS-CoV-2 nucleocapsid RNA binding domain by human monoclonal antibodies<br><br>Youngchang Kim, Natalia Maltseva, Christine Tesar , Robert Jedrzejczak , Michael Endres , Heng Ma , Haley L Dugan , Christopher T Stamper , Changsoo Chang , Lei Li , Siriruk Changrob , Nai-Ying Zheng , Min Huang , Arvind Ramanathan , Patrick Wilson , Karolina Michalska , Andrzej Joachimiak ,<br><br>(2024) iScience 27(2):108976.

15.	7STR	X-Ray 1.50 Å	SARS-CoV-2 N-NTD, aa 47-172	N-NTD-Human Fab S24-1063 complex	N-NTD monomer	Epitopes recognition of SARS-CoV-2 nucleocapsid RNA binding domain by human monoclonal antibodies<br><br>Youngchang Kim, Natalia Maltseva, Christine Tesar , Robert Jedrzejczak , Michael Endres , Heng Ma , Haley L Dugan , Christopher T Stamper , Changsoo Chang , Lei Li , Siriruk Changrob , Nai-Ying Zheng , Min Huang , Arvind Ramanathan , Patrick Wilson , Karolina Michalska , Andrzej Joachimiak ,<br><br>(2024) iScience 27(2):108976.

16.	7N3C	X-Ray 1.82 Å	SARS-CoV-2 N-NTD, aa 47-173	N-NTD-Human Fab S24-202 complex	N-NTD monomer	Epitopes recognition of SARS-CoV-2 nucleocapsid RNA binding domain by human monoclonal antibodies<br><br>Youngchang Kim, Natalia Maltseva, Christine Tesar , Robert Jedrzejczak , Michael Endres , Heng Ma , Haley L Dugan , Christopher T Stamper , Changsoo Chang , Lei Li , Siriruk Changrob , Nai-Ying Zheng , Min Huang , Arvind Ramanathan , Patrick Wilson , Karolina Michalska , Andrzej Joachimiak ,<br><br>(2024) iScience 27(2):108976.

17.	7N3D	X-Ray 1.53 Å	SARS-CoV-2 N-NTD, aa 49-171	N-NTD-Human Fab S24-1564 complex	N-NTD monomer	Epitopes recognition of SARS-CoV-2 nucleocapsid RNA binding domain by human monoclonal antibodies<br><br>Youngchang Kim, Natalia Maltseva, Christine Tesar , Robert Jedrzejczak , Michael Endres , Heng Ma , Haley L Dugan , Christopher T Stamper , Changsoo Chang , Lei Li , Siriruk Changrob , Nai-Ying Zheng , Min Huang , Arvind Ramanathan , Patrick Wilson , Karolina Michalska , Andrzej Joachimiak ,<br><br>(2024) iScience 27(2):108976.
18.	7VBD	X-Ray 1.94 Å	SARS-CoV-2 N-NTD, aa 49-171	N-NTD apo	N-NTD tetramer	To be published.
19.	7SD4	Solid-State NMR	SARS-CoV-2 N-NTD, aa 39-174	N-NTD apo	N-NTD ensemble	Atomic-Resolution Structure of SARS-CoV-2 Nucleocapsid Protein N-Terminal Domain.<br><br>Sucharita Sarkar , Brent Runge , Ryan W Russell , Kumar Tekwani Movellan , Daniel

						Calero , Somayeh Zeinalilathori , Caitlin M Quinn , Manman Lu , Guillermo Calero , Angela M Gronenborn , Tatyana Polenova , (2022) J Am Chem Soc 144: 10543-10555
20.	6YI3	Solution NMR	SARS-CoV-2 N-NTD, aa 44-180	N-NTD apo	N-NTD ensemble	Structural basis of RNA recognition by the SARS-CoV-2 nucleocapsid phosphoprotein<br>Dhurvas Chandrasekaran Dinesh , Dominika Chalupska , Jan Silhan , Eliska Koutna , Radim Nencka , Vaclav Veverka , Evzen Boura ,<br>(2020) PLoS Pathog 16: e1009100-e1009100
21.	7ACT	Solution NMR	SARS-CoV-2 N-NTD, aa 44-180	N-NTD – ssRNA complex, ssRNA sequence (5'-UCUCUAAACG-3')	N-NTD-ssRNA ensemble	Structural basis of RNA recognition by the SARS-CoV-2 nucleocapsid phosphoprotein<br>Dhurvas Chandrasekaran Dinesh , Dominika Chalupska , Jan Silhan , Eliska Koutna , Radim Nencka , Vaclav Veverka , Evzen Boura ,

						(2020) PLoS Pathog 16: e1009100-e1009100
22.	7ACS	Solution NMR	SARS-CoV-2 N-NTD, aa 44-180	N-NTD – dsRNA complex, dsRNA sequence (5'-CACUGAC-3' and 5'-GUCAGUG-3')	N-NTD-ssRNA ensemble	Structural basis of RNA recognition by the SARS-CoV-2 nucleocapsid phosphoprotein<br>Dhurvas Chandrasekaran Dinesh , Dominika Chalupska , Jan Silhan , Eliska Koutna , Radim Nencka , Vaclav Veverka , Evzen Boura<br>(2020) PLoS Pathog 16: e1009100-e1009100
23	8TFD	X-Ray 1.55 Å	SARS-CoV-2 N-NTD, aa 46-174	N-NTD-DNA - aptamer complex, DNA sequence (5′-TCGGACATCGGATTGTCTGA-3′)	N-NTD monomer with DNA aptamer	A compact stem-loop DNA aptamer targets a uracil-binding pocket in the SARS-CoV-2 nucleocapsid RNA-binding domain<br>Morgan A Esler , Christopher A Belica , Joseph A Rollie , William L Brown , Seyed Arad Moghadasi , Ke Shi , Daniel A Harki, Reuben S Harris, Hideki Aihara<br>(2024) Nucleic Acids Res 52(21):13138-13151
